# Association of prenatal psychological distress and postpartum depression with varying physical activity intensity: Japan Environment and Children’s Study (JECS)

**DOI:** 10.1038/s41598-020-63268-1

**Published:** 2020-04-14

**Authors:** Ryoko Susukida, Kentaro Usuda, Kei Hamazaki, Akiko Tsuchida, Kenta Matsumura, Daisuke Nishi, Hidekuni Inadera, Japan Environment and Children’s Study (JECS) Group, Michihiro Kamijima, Shin Yamazaki, Yukihiro Ohya, Reiko Kishi, Nobuo Yaegashi, Koichi Hashimoto, Chisato Mori, Shuichi Ito, Zentaro Yamagata, Takeo Nakayama, Hiroyasu Iso, Masayuki Shima, Youichi Kurozawa, Narufumi Suganuma, Koichi Kusuhara, Takahiko Katoh

**Affiliations:** 1 Department of Public Health, Faculty of Medicine, University of Toyama, Toyama, Japan; 2 Department of Mental Health Policy, National Institute of Mental Health, National Center of Neurology and Psychiatry, Kodaira, Tokyo, Japan; 3 Department of Mental Health, Johns Hopkins Bloomberg School of Public Health, Baltimore, USA; 4 Toyama Regional Center for JECS, University of Toyama, Toyama, Japan; 5 Department of Mental Health, Graduate School of Medicine, The University of Tokyo, Tokyo, Japan; 6 Graduate School of Medical Sciences Department of Occupational and Environmental Health, Nagoya City University, 1 Kawasumi, Mizuho-cho, Mizuho-ku, Nagoya, Aichi 467-8601 Japan; 7 National Institute for Environmental Studies, Tsukuba, Japan; 8 National Center for Child Health and Development, Tokyo, Japan; 9 Hokkaido University, Sapporo, Japan; 10 Tohoku University, Sendai, Japan; 11 Fukushima Medical University, Fukushima, Japan; 12 Chiba University, Chiba, Japan; 13 Yokohama City University, Yokohama, Japan; 14 University of Yamanashi, Chuo, Japan; 15 Kyoto University, Kyoto, Japan; 16 Osaka University, Suita, Japan; 17 Hyogo College of Medicine, Nishinomiya, Japan; 18 Tottori University, Yonago, Japan; 19 Kochi University, Nankoku, Japan; 20 University of Occupational and Environmental Health, Kitakyushu, Japan; 21 Kumamoto University, Kumamoto, Japan

**Keywords:** Epidemiology, Risk factors

## Abstract

Evidence is mixed on the associations between physical activity during pregnancy and perinatal depression, and it is limited for different physical activity intensities. Data for 92,743 pregnant women from the Japan Environment and Children’s Study were analyzed in this study. Psychological distress during pregnancy was assessed as moderate or severe using the Kessler Psychological Distress Scale (K6 5–12 and ≥13, respectively). Postpartum depression was assessed using the Edinburgh Postpartum Depression Scale (EPDS; cut-off score 9). Women with only light physical activity had significantly lower odds of psychological distress during pregnancy than those with no physical activity (K6 5–12: adjusted odds ratio [AOR] 0.86, 95% confidence interval [95%CI] 0.82, 0.90; K6 ≥ 13: AOR 0.64, 95%CI 0.58, 0.72). Women with a combination of light, moderate and vigorous physical activity had significantly higher odds of psychological distress during pregnancy (K6 5–12: AOR 1.32, 95%CI 1.18, 1.48; K6 ≥ 13: AOR 1.45, 95%CI 1.16, 1.81) and depression after childbirth (EPDS ≥ 9: AOR 1.42, 95%CI 1.24, 1.61). Physical activity intensity should be considered when assessing psychological distress risk during pregnancy and depression risk after delivery. Future research should evaluate specific physical activity programs with optimal intensity for pregnant women to prevent and treat their psychological distress and depression.

## Introduction

The risk of depression is substantially elevated throughout the course of pregnancy^1,2^. Studies have estimated the prevalence of depression of mild or greater severity to be as high as 20% during pregnancy^3^ and 13% after childbirth^4^. Psychological distress, which is a state of emotional discomfort accompanied by symptoms of anxiety and depression^5^, is also seen during pregnancy; severe psychological distress is seen in 3.1%-3.4% of cases^6^ and moderate psychological distress in 28.6%-34.6%^7^. It has also been reported that depression during pregnancy is associated with the potential increased risk of adverse birth outcomes such as preterm labour and low birth weight^8–11^. Furthermore, depression after childbirth is known to compromise mothers’ everyday functioning, such as self-care, as well as bonding and healthy interactions with their children^12^.

Physical activity is the most commonly recommended form of lifestyle modification for treating depression during pregnancy^13^, although evidence is mixed for the association between physical activity during pregnancy and depression over the course of pregnancy. While increasing numbers of studies are reporting that physical activity is associated with lower depression^14–16^, there are also studies reporting no associations between physical activity and depression in pregnant women^17–19^ and a few studies reporting increased risk of depression associated with physical activity in pregnant women^20,21^. Most of these previous studies focused on cross-sectional associations between physical activity and depression during pregnancy and only a limited number of studies have examined longitudinal associations between physical activity during pregnancy and postpartum depression^21,22^.

Little is also understood about the optimal intensity of physical activity that would be potentially beneficial for psychological distress and depression. According to a review by Teychenne and York^23^, most studies either did not assess how the associations between varying intensities of physical activity and mental health outcomes differ or they focused on the psychological effects of only moderate to vigorous physical activity. As such, there is no clear consensus on the optimal intensity of physical activity for lowering the risk of depression over the course of pregnancy.

Additionally, previous studies, with a few exceptions^17,18^, have often assessed both cross-sectional and longitudinal associations between physical activity and depression without controlling for baseline physical activity prior to pregnancy—this factor could greatly affect women’s engagement in physical activity during pregnancy and their mental health outcomes. Downs *et al*.^18^ found that physical activity prior to pregnancy, but not during pregnancy, was significantly associated with lower risk of postpartum depressive symptoms. Ersek and Brunner Huber^17^ reported that only women who were physically active both prior to and during pregnancy had lower risk of depressive symptoms compared with those who were not physically active prior to and during pregnancy. Clearly, there is a need for more research assessing whether physical activity during pregnancy can actually be beneficial for the psychological well-being of pregnant women while also controlling for their physical activity prior to pregnancy.

This study assessed associations between varying combinations of physical activity at different intensities during pregnancy and psychological distress and depression in a large sample of pregnant women drawn from the Japan Environment and Children’s Study (JECS). We examined not only the cross-sectional association between physical activity and psychological distress during pregnancy but also the longitudinal association between physical activity during pregnancy and postpartum depression while also controlling for potential confounding factors, including physical activity prior to pregnancy.

## Methods

### Study sample

This study used data from pregnant women residing in Japan who participated in the JECS, a large-scale ongoing national birth cohort study launched by the Japan Ministry of the Environment in January 2011^24,25^. The JECS collects a wide range of information on health and environmental factors as they relate to pregnant women and their children with the aim of identifying factors that are critical to child development and to the well-being of mothers and their children. Over 100,000 pregnant women from 15 different regions in Japan were recruited between 2011 and 2014, and the characteristics of the mothers and children are almost identical to those reported during same period by Vital Statistics of Japan^25^. The children are currently being followed using questionnaire surveys until they turn 13 years of age.

**Figure 1:**
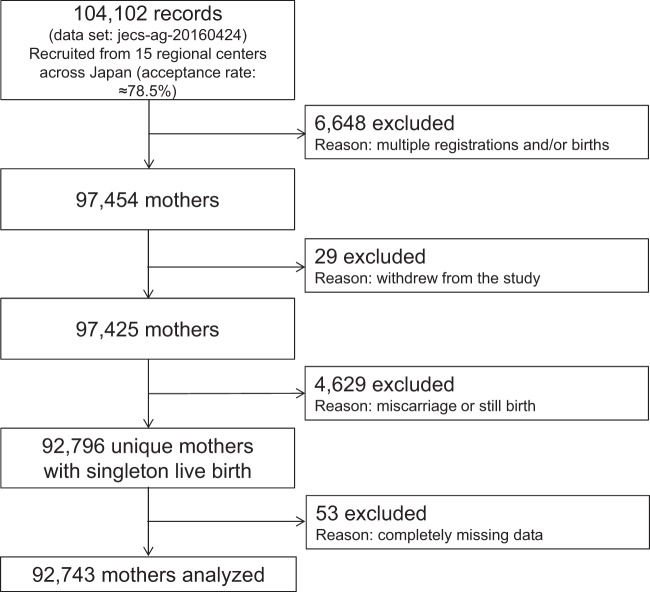
Participant flow diagram.

In this study, we used the jecs-ag-20160424 dataset released in June 2016 and the allbirth_revice001_ver001 dataset released in October 2016. From a total of 92,796 pregnant women, we excluded 53 whose information was completely missing for the selected variables used in this study (see Measures). This left data for 92,743 women for analysis in this study. Figure 1 shows the flowchart of participant recruitment and selection.

The authors assert that all procedures contributing to this work comply with the ethical standards of the relevant national and institutional committees on human experimentation and with the Helsinki Declaration of 1975, as revised in 2008. All procedures involving human patients were approved by the Ministry of the Environment’s Institutional Review Board on Epidemiological Studies (100910001), the ethics committees of all participating institutions, and the Ethics Committee, University of Toyama (R2017163). Written informed consent was obtained from all patients.

### Measures

The outcome variables in this study were 1) prenatal psychological distress (mid-late pregnancy) and 2) postpartum depression. Prenatal psychological distress was assessed on two occasions using the Kessler Psychological Distress Scale (K6):^26,27^ once during early pregnancy, which was used as a covariate, and once during mid-late pregnancy, which was used as the outcome. Postpartum depression was assessed using the Edinburgh Postpartum Depression Scale (EPDS) 1 month after childbirth^28^. Moderate psychological distress during pregnancy was defined as a K6 score of 5–12, and severe psychological distress during pregnancy was defined as a K6 score of 13 or higher^29,30^. Postpartum depression was defined by an EPDS score of 9 or higher^31,32^.

The main explanatory variable was a combination of varying intensities of physical activity during pregnancy. Intensity of physical activity during pregnancy was assessed with the translated short version of the International Physical Activity Questionnaire (IPAQ)^33,34^. Intensity of physical activity was assessed during mid-late pregnancy when a prenatal measure of psychological distress was also administered at hospitals or clinics affiliated with regional health centres. Three different intensities of physical activity (vigorous, moderate and light) were assessed in a self-reported questionnaire. The following questions were used to assess the different intensities of physical activity: for vigorous activity, “Within the past week, did you engage in vigorous physical activity, including carrying or moving heavy objects, biking on steep hills, jogging, or playing singles at tennis?” For moderate activity, “Within the past week, did you engage in moderate physical activity, including carrying or moving light objects, playing and running with children, swimming slowly, playing doubles at tennis, or playing golf without using a golf cart? Please do not include walking as a form of moderate physical activity”. For light activity, “Within the past week, did you engage in light physical activity, such as walking more than 10 minutes? Please include all forms of walking including walking at work or in daily life and walking as a form of activity or hobby”. Each variable was recorded as a dummy variable (yes = 1; no = 0). These variables were not mutually exclusive, meaning that each study participant could engage in any combinations of the three different intensities of physical activity. Therefore, we created a categorical variable of physical activity during pregnancy (no activity = 0; light activity only = 1; moderate activity only = 2; vigorous activity only = 3; light and moderate activity = 4; light and vigorous activity = 5; moderate and vigorous activity = 6; light, moderate and vigorous activity = 7).

The analyses were adjusted for a number of potential confounders that were found in past research to be associated with pregnancy-related psychological distress and depression^17,35–39^. Covariates included physical activity prior to pregnancy (three dichotomous variables indicating whether JECS participants engaged in light, moderate and vigorous physical activity), psychological distress measured by the K6 scale during early pregnancy (no, moderate or severe distress), body mass index (BMI; early pregnancy), smoking status (early pregnancy), lifetime gestational diabetes, age (continuous), educational attainment (high school or lower, junior college or 4-year college or higher), income (<¥4,000,000, ¥4,000,000–¥6,000,000 or ≥¥6,000,000), marital status (married, single or divorced/widowed), work status (working or not working) and previous childbirth experience (yes or no).

### Data analysis

We first descriptively assessed the distribution of physical activity intensities and other JECS participant characteristics according to different levels of prenatal psychological distress and postpartum depression. Pearson’s chi-squared test was used to determine if the study participants’ characteristics differed according to level of psychological distress. Next, we estimated a multinomial logistic regression model with the ordinal outcome variable of prenatal psychological distress (0: no distress, 1: moderate distress and 2: severe distress) as a dependent variable and the categorical variable of physical activity as a main explanatory variable while controlling for covariates. Finally, we estimated a multivariable logistic regression model with the binary outcome variable of postpartum depression (0: no distress and 1: distress) as a dependent variable and the categorical variable of physical activity as a main explanatory variable while controlling for covariates. Because 26.9% of the study sample (25,025/92,743) had at least one missing variable, we performed multiple imputation and conducted regression models with five imputed datasets. Data were analyzed using STATA SE version 14 (StataCorp LP, College Station, TX). The detailed patterns of missing data are described in Supplementary Table S1.

## Results

### Descriptive analysis

Table 1 shows comparisons of sample characteristics across subgroups of mothers with and without prenatal psychological distress and postpartum depression. Except for the baseline prevalence of lifetime gestational diabetes, the distributions of all the other variables differed significantly across subgroups of women with different levels of psychological distress in mid-late pregnancy. During mid-late pregnancy, women with K6 5–12 were more likely to engage in light (71.37% vs 70.59%), moderate (41.91% vs 39.65%) and vigorous (17.65% vs 14.87%) physical activity than women with K6 < 5. Similarly, women with K6 ≥ 13 were more likely to engage in light (71.95% vs 70.59%), moderate (42.31% vs 39.65%), and vigorous (22.70% vs 14.87%) physical activity than women with K6 < 5.

**Table 1:** Participants’ characteristics.

	All N = 92,796	Mid-late pregnancy, K6 (<5) n = 63,943	Mid-late pregnancy, K6 (5–12) n = 23,337	Mid-late pregnancy, K6 (≥13) n = 2,971	P-value Pearson χ^2^ Test	1 month after delivery, EPDS (<9) n = 76,332	1 month after delivery, EPDS (≥9) n = 12,818	P-value Pearson χ^2^ Test
	%	%	%	%		%	%	
**Baseline mental health (early pregnancy)**								
K6 (<5)	67.74	81.20	37.91	12.56	p < 0.01	73.54	34.09	p < 0.01
K6 (5–12) moderate distress	28.72	18.01	55.34	50.53		24.67	52.07	
K6 (≥13) severe distress	3.55	0.79	6.74	36.90		1.79	13.53	
**Baseline physical health (early pregnancy)**								
BMI (18.5–24.9)	74.4	75.11	73.00	69.27	p < 0.01	74.89	72.02	p < 0.01
Lifetime gestational diabetes	0.69	0.67	0.76	0.88	p = 0.16	0.68	0.76	p = 0.31
Smoking	4.81	3.87	6.27	12.01	p <0.01	4.19	7.14	p < 0.01
**Baseline physical activity (prior to pregnancy)**^**a**^								
Light activity	70.82	70.59	71.37	71.95	p = 0.04	70.78	70.96	p = 0.69
Moderate activity	40.29	39.65	41.91	42.31	p < 0.01	40.57	39.40	p = 0.01
Vigorous activity	15.85	14.87	17.65	22.70	p < 0.01	15.29	18.98	p < 0.01
**Physical activity during pregnancy (second trimester)**^**a**^								
Light activity	71.75	72.24	71.57	69.19	p < 0.01	71.82	72.94	p = 0.01
Moderate activity	24.54	23.67	26.55	27.12	p < 0.01	24.40	25.36	p = 0.02
Vigorous activity	3.32	2.68	4.55	7.33	p < 0.01	3.05	4.79	p < 0.01
**Demographic characteristics**								
Age (≥30 years old)	59.50	62.07	54.56	46.28	p < 0.01	60.76	53.52	p < 0.01
Educational attainment (university degree or higher)	21.72	23.02	19.19	13.84	p < 0.01	22.59	17.60	p < 0.01
Household income (≥ ¥6,000,000)	26.88	28.56	23.53	16.17	p < 0.01	27.90	21.33	p < 0.01
Marital status (Married)	95.43	96.52	93.49	88.81	p < 0.01	96.08	92.42	p < 0.01
Work status (Working)	63.18	64.05	61.69	55.95	p < 0.01	63.61	61.84	p < 0.01
Prior childbirth	57.46	58.24	55.57	54.58	p < 0.01	59.34	46.06	p < 0.01

EPDS: Edinburgh Postpartum Depression Scale; K6: Kessler Psychological Distress Scale.

^a^Categories of intensity of physical activity were not mutually exclusive, so percentages do not necessarily add up to 100%.

Distributions of all variables except for the baseline prevalence of lifetime gestational diabetes and baseline frequency of light physical activity differed significantly across subgroups of women with different levels of postpartum depression. After childbirth, women with EPDS ≥ 9 were less likely to engage in moderate physical activity (39.40% vs 40.57%) and more likely to engage in vigorous physical activity (22.70% vs 14.87%) than women with EPDS < 9.

### Prenatal psychological distress

As shown in Table 2, after adjusting for physical activity prior to pregnancy, mental health condition (early pregnancy), BMI (early pregnancy), smoking status (early pregnancy), lifetime gestational diabetes, age, education, income, marital status, work status and previous childbirth experience, compared with women without any physical activity during pregnancy, women with only light activity during pregnancy had 14% lower odds of moderate prenatal psychological distress (adjusted odds ratio [AOR] 0.86, 95% confidence interval [95%CI] 0.82, 0.90), women with a combination of light and moderate physical activity during pregnancy had 7% lower odds of moderate prenatal psychological distress (AOR 0.93, 95%CI 0.88, 0.99), while women with a combination of light and vigorous physical activity had 25% higher odds of moderate prenatal psychological distress (AOR 1.25, 95%CI 1.02, 1.53) and women with a combination of moderate and vigorous physical activity during pregnancy had 35% higher odds of moderate prenatal psychological distress (AOR 1.35, 95%CI 1.02, 1.79). Finally, women with a combination of light, moderate and vigorous physical activity during pregnancy had 32% higher odds of moderate prenatal psychological distress (AOR 1.32, 95%CI 1.18, 1.48) than those without any physical activity during pregnancy.

**Table 2:** Multinomial logistic regression analysis of the associations of prenatal moderate and severe psychological distress with varying intensities of physical activity during pregnancy.

Physical activity during pregnancy	K6 (5–12)			K6 (≥13)		
	Cases (n)	AOR	95%CI	Cases (n)	AOR	95%CI
No activity	3,996	1.00	—	522	1.00	—
Light activity only	8,308	0.86**	0.82, 0.90	905	0.64**	0.58, 0.72
Moderate activity only	792	0.95	0.87, 1.03	82	0.63**	0.50, 0.79
Vigorous activity only	36	1.29	0.86, 1.92	2	0.67	0.25, 1.85
Light & moderate activity	3,161	0.93*	0.88, 0.99	355	0.69**	0.60, 0.79
Light & vigorous activity	128	1.25*	1.02, 1.53	29	1.42	0.96, 2.12
Moderate & vigorous activity	75	1.35*	1.02, 1.79	6	1.12	0.62, 2.03
Light, moderate & vigorous activity	500	1.32**	1.18, 1.48	108	1.45**	1.16, 1.81

AOR: adjusted odds ratios; 95%CI: 95% confidence intervals; K6 = Kessler Psychological Distress Scale.

Notes: Adjusted for physical activity prior to pregnancy, mental health condition (K6) during early pregnancy, body mass index (early pregnancy), smoking status (early pregnancy), lifetime gestational diabetes, age, education, income, marital status, work status, and previous childbirth experience. *p < 0.05. **p < 0.01.

In terms of severe prenatal psychological distress, compared with women without any physical activity during pregnancy, women with only light physical activity during pregnancy had 36% lower odds of severe prenatal psychological distress (AOR 0.64, 95%CI 0.58, 0.72), women with only moderate physical activity during pregnancy had 37% lower odds of severe prenatal psychological distress (AOR 0.63, 95%CI 0.50, 0.79), and women with a combination of light and moderate physical activity had 31% lower odds of severe prenatal psychological distress (AOR 0.69, 95%CI 0.60, 0.79) In contrast, women with a combination of light, moderate and vigorous physical activity had 45% higher odds of severe prenatal psychological distress (AOR 1.45, 95%CI 1.16, 1.81) than those without any physical activity during pregnancy.

### Postpartum depression

As shown in Table 3, after adjusting for physical activity prior to pregnancy, psychological distress (early pregnancy), BMI (early pregnancy), smoking status (early pregnancy), lifetime gestational diabetes, age, education, income, marital status, work status and previous childbirth experience, women with a combination of light, moderate and vigorous activity during pregnancy had 42% higher odds of postpartum depression (AOR, 1.42; 95%CI, 1.24, 1.61) than those without any physical activity during pregnancy. Except for this combination of light, moderate and vigorous activity, no statistically significant association was found between the physical activity categories and postpartum depression.

**Table 3:** Multivariable logistic regression analysis of the associations of postpartum depression with varying intensities of physical activity during pregnancy.

Physical activity during pregnancy	EPDS (≥9)		
	Cases (n)	AOR	95%CI
No activity	2,078	1.00	—
Light activity only	4,734	0.99	0.94, 1.04
Moderate activity only	376	0.97	0.87, 1.08
Vigorous activity only	16	1.25	0.74, 2.11
Light & moderate activity	1,639	1.07	0.99, 1.15
Light & vigorous activity	75	1.21	0.97, 1.51
Moderate & vigorous activity	30	1.12	0.79, 1.57
Light, moderate & vigorous activity	300	1.42**	1.24, 1.61

AOR: adjusted odds ratios; 95%CI: 95% confidence intervals; EPDS: Edinburgh Postpartum Depression Scale

Notes: Adjusted for physical activity prior to pregnancy, mental health condition (K6) during early pregnancy, body mass index (early pregnancy), smoking status (early pregnancy), lifetime gestational diabetes, age, education, income, marital status, work status, and previous childbirth experience. *p < 0.05. **p < 0.01.

## Discussion

The associations between physical activity and prenatal psychological distress and postpartum depression varied in this study depending on which combinations of different intensities of physical activity the women engaged in during pregnancy. Most commonly, those women who engaged in only light physical activity during pregnancy were significantly less likely to have psychological distress during pregnancy, whereas those women who engaged in a combination of all light, moderate and vigorous physical activity during pregnancy were more likely to have psychological distress during pregnancy and depression after childbirth. Most studies have concluded that regular physical exercise during pregnancy has a beneficial impact on psychological health^40^, but our results are somewhat similar to those of other studies using general population data^41,42^. The present study is one of the few studies to show that different combinations of varying intensities of physical activity had different patterns of associations with psychological outcomes among pregnant women.

There might be various reasons for the differences we found in the degree of association between physical activity and psychological distress and depression according to different combinations of varying intensities of physical activity, and we were not able to empirically examine them in this study. For example, it is known that women with higher socioeconomic status are more likely to engage in voluntary activity programs such as jogging, aerobics, dance classes and team sports than those with lower socioeconomic status^43^. Women who voluntarily engaged in these physical activity programs might have had better psychological status during pregnancy and after childbirth in the sample used in the present study, but future studies are needed to explore the reasons and motivations behind engagement in physical activity during pregnancy. As another example, those pregnant women who decided or needed to keep working during pregnancy might have had to perform physical activities during worktime in addition to their domestic responsibilities, which could have been associated with higher psychological distress. This is plausible given that women who engaged in a combination of light, moderate and vigorous physical activity during pregnancy were more likely to be working during pregnancy than those women who engaged in light physical activity only (71.6% vs 59.4%, p < 0.01). They were also more likely to be in industries such as construction and logistics (1.63% vs 0.90%, p < 0.01) where employees may undertake physical tasks such as lifting and moving objects more frequently than employees in other administrative-oriented industries. Future studies should examine the patterns and characteristics of physical activity undertaken by pregnant women in different occupations.

In interpreting the results of the present study, several limitations should be taken into consideration. First, the observational nature of the data did not allow us to draw causal inferences of the relationship between physical activity and psychological distress and depression. Even though a strength of this study was that we could control for pre-pregnancy physical activity, baseline psychological distress and many other potential confounding factors, exposure to physical activity was far from random in the experimental study settings, and it is possible that the level of physical activity was influenced by psychological distress. Second, those with baseline psychological distress might have been more likely to refuse to participate in the study or more likely to drop out of the study, which might have resulted in selection bias. Nonetheless, more than 30% of the participants in the sample had moderate or greater psychological distress at baseline. Additionally, we applied multiple imputation techniques to handle missing data based on the observable variables in the study sample. Third, the JECS data did not contain information about specific domains of physical activity. A few studies have reported that the associations between physical activity and mental health outcomes differed by domain of physical activity. For instance, Demissie *et al*.^20^ assessed five domains (occupational, recreational, child and adult care, indoor household activity and outdoor household activity) in 550 pregnant women in North Carolina and found that only those who engaged in moderate to vigorous physical activity in adult- or childcare had significantly higher odds of postpartum depressive symptoms than those who did not participate in any moderate to vigorous physical activity. The positive association between a combination of light, moderate and vigorous physical activity and prenatal psychological distress and postpartum depression that we found in the present study may reflect the fact that those pregnant women who engaged in various intensities of task-oriented physical activities out of necessity (e.g., to care for family members) might have been at increased risk of developing depression. Future studies should explore specific domains of physical activity during pregnancy to better understand the potential mechanisms through which certain intensities of physical activity could have either a beneficial or harmful impact on psychological well-being over the course of pregnancy. Fourth, retrospective recall of physical activity prior to and during pregnancy may be subject to recall bias. Studies have shown that self-reported physical activity during pregnancy alone tends to overestimate activity status compared with self-reported physical activity during pregnancy confirmed by an objective activity monitor^44^. Future studies should incorporate the use of such an instrument to increase the accuracy of the measurement of physical activity during pregnancy and to enable real-time collection of environmental information (e.g., seasonality, weather conditions, temperature, and geographical context) that could influence the level of physical activity during pregnancy. Fifth, even though measurement properties of the IPAQ have been studied in pregnant women across multiple countries, no validation studies have been conducted with Japanese pregnant women. Future research should assess the validity and reliability of the IPAQ in this population. Lastly, although our results which were estimated from a large dataset on pregnant women in Japan could be mostly applicable to the entire population of pregnant women in Japan, our findings may not be necessarily directly applicable to pregnant women in other countries with different cultural and socioeconomic backgrounds.

Despite these limitations, our study provides clear evidence that the associations between physical activity and prenatal psychological distress and postpartum depression differ depending on which combinations of varying intensities of physical activity women engage in during pregnancy. Future work should examine whether there is variation in the magnitude of these associations based on the women’s baseline characteristics, such as pre-pregnancy health issues and previous childbirth experience. Future studies should also explore the contexts and domains of engagement in physical activity to better understand in what contexts and at what levels of physical activity intensity could be beneficial or harmful to the mental well-being of pregnant women. Further research in this area could also lead to evidence-based health guidelines of specific activity programs with optimal intensity for pregnant women to facilitate prevention and treatment of psychological distress and depression.

## Supplementary information

Supplementary Dataset 3.

## Data Availability

Data are unsuitable for public deposition due to ethical restrictions and Japan’s legal framework. It is prohibited by the Act on the Protection of Personal Information (Act No. 57 of 30 May 2003, amendment on 9 September 2015) to publicly deposit data containing personal information. Ethical Guidelines for Epidemiological Research enforced by the Japan Ministry of Education, Culture, Sports, Science and Technology and the Ministry of Health, Labour and Welfare also restricts the open sharing of the epidemiological data. All inquiries about access to data should be sent to: jecs-en@nies.go.jp. The person responsible for handling enquiries sent to this e-mail address is Dr Shoji F. Nakayama, JECS Programme Office, National Institute for Environmental Studies.
