# Supplementary material for: Association of prenatal psychological distress and postpartum depression with varying physical activity intensity: Japan Environment and Children’s Study (JECS)
Source: Sci Rep. 2020 Apr 14;10:6390. doi: 10.1038/s41598-020-63268-1 (PMC7156653; doi:10.1038/s41598-020-63268-1)
Supplement: Supplementary file 1 — Supplementary Dataset 3. [file 41598_2020_63268_MOESM1_ESM.docx]

**Association of prenatal psychological distress and postpartum depression with varying physical activity intensity: Japan Environment and Children’s Study (JECS)**

Ryoko Susukida^1, 2, 3^, Kentaro Usuda^1, 2^, Kei Hamazaki^1, 4, *^, Akiko Tsuchida^1, 4^, Kenta Matsumura^4^, Daisuke Nishi^1, 1, 5^, Hidekuni Inadera^1, 4^, Japan Environment and Children’s Study (JECS) Group ^**^(Nominated consortia representative: Michihiro Kamijima^6^)

^1^Department of Public Health, Faculty of Medicine, University of Toyama, Toyama, Japan

^2^Department of Mental Health Policy, National Institute of Mental Health, National Center of Neurology and Psychiatry, Kodaira, Tokyo, Japan

^3^Department of Mental Health, Johns Hopkins Bloomberg School of Public Health, Baltimore, USA

^4^Toyama Regional Center for JECS, University of Toyama, Toyama, Japan

^5^Department of Mental Health, Graduate School of Medicine, The University of Tokyo, Tokyo, Japan

^6^Graduate School of Medical Sciences Department of Occupational and Environmental Health, Nagoya City University, 1 Kawasumi, Mizuho-cho, Mizuho-ku, Nagoya, Aichi 467-8601, Japan. E-mail: jecscore@nies.go.jp

^**^Members of the JECS Group are listed at the end of the manuscript.

^*^**Corresponding author**: Kei Hamazaki

Department of Public Health

Faculty of Medicine, University of Toyama

2630 Sugitani, Toyama-shi, Toyama 930-0194, Japan

Telephone: +81-76-434-7275

E-mail: keihama@med.u-toyama.ac.jp

**Running head**: Distress and physical activity during pregnancy

**Clinical trial registration number**: UMIN000030786

<https://upload.umin.ac.jp/cgi-open-bin/ctr/ctr_view.cgi?recptno=R000035091>

**Supplementary Table S1 Number and percentage of cases with missing values for each covariate**

|  | Valid n | Missing n | % Missing n |
| --- | --- | --- | --- |
| Total | 67,718 | 25,025 | 26.9 |
| K6 (early pregnancy) | 90,405 | 2,391 | 2.6 |
| K6 (during pregnancy) | 90,251 | 2,545 | 2.7 |
| EPDS (after childbirth) | 89,150 | 3,646 | 3.9 |
| Body mass index | 90,021 | 2,775 | 3.0 |
| Lifetime gestational diabetes | 91,502 | 1,294 | 1.4 |
| Smoking | 90,815 | 1,981 | 2.1 |
| Physical activity (baseline) | 84,663 | 8,133 | 8.8 |
| Physical activity (during pregnancy) | 87,916 | 4,880 | 5.3 |
| Age | 91,488 | 1,308 | 1.4 |
| Educational attainment | 90,557 | 2,239 | 2.4 |
| Household income | 84,497 | 8,299 | 8.9 |
| Marital status | 91,090 | 1,706 | 1.8 |
| Work status | 88,433 | 4,363 | 4.7 |
| Prior childbirth | 90,439 | 2,357 | 2.5 |

K6, Kessler Psychological Distress Scale; EPDS, Edinburgh Postpartum Depression Scale

**Supplementary Table S2. Multinomial logistic regression analysis of the associations of prenatal moderate and severe psychological distress with varying intensities of physical activity during pregnancy (n = 67,718, complete cases only)**

|  | K6 (5-12) | | K6 (≥ 13) | |
| --- | --- | --- | --- | --- |
| Physical activity during pregnancy | AOR | 95%CI | AOR | 95%CI |
| No activity | 1.00 | -- | 1.00 | -- |
| Light activity only | 0.85** | 0.81, 0.90 | 0.68** | 0.59, 0.77 |
| Moderate activity only | 0.93 | 0.85, 1.03 | 0.65** | 0.50, 0.84 |
| Vigorous activity only | 1.49 | 0.93, 2.39 | 0.40 | 0.09, 1.77 |
| Light & moderate activity | 0.93* | 0.87, 0.99 | 0.69** | 0.59, 0.82 |
| Light & vigorous activity | 1.24 | 0.97, 1.58 | 1.70* | 1.07, 2.71 |
| Moderate & vigorous activity | 1.37 | 0.99, 1.89 | 0.82 | 0.34, 1.96 |
| Light, moderate & vigorous activity | 1.35** | 1.18, 1.55 | 1.67** | 1.28, 2.18 |
| AOR: adjusted odds ratios; 95%CI: 95% confidence intervals; K6 = Kessler Psychological Distress Scale  Notes: Adjusted for physical activity prior to pregnancy, mental health condition (K6) during early pregnancy, body mass index (early pregnancy), smoking status (early pregnancy), lifetime gestational diabetes, age, education, income, marital status, work status, and previous childbirth experience. *p < 0.05. **p < 0.01. | | | | |

**Supplementary Table S3. Multivariable logistic regression analysis of the associations of postpartum depression with varying intensities of physical activity during pregnancy (n = 67,718, complete cases only)**

|  | EPDS (≥ 9) | |
| --- | --- | --- |
| Physical activity during pregnancy | AOR | 95%CI |
| No activity | 1.00 | -- |
| Light activity only | 1.00 | 0.94, 1.07 |
| Moderate activity only | 0.95 | 0.84, 1.08 |
| Vigorous activity only | 1.00 | 0.55, 1.79 |
| Light & moderate activity | 1.07 | 0.98, 1.16 |
| Light & vigorous activity | 1.19 | 0.90, 1.57 |
| Moderate & vigorous activity | 1.02 | 0.68, 1.54 |
| Light, moderate & vigorous activity | 1.48** | 1.27, 1.73 |
| AOR: adjusted odds ratio; 95%CI: 95% confidence intervals; EPDS: Edinburgh Postpartum Depression Scale  Notes: Adjusted for physical activity prior to pregnancy, mental health condition (K6) during early pregnancy, body mass index (early pregnancy), smoking status (early pregnancy), lifetime gestational diabetes, age, education, income, marital status, work status, and previous childbirth experience. *p < 0.05. **p < 0.01. | | |

**Consortia**

**The Japan Environment & Children’s Study (JECS) Group**

Members of the JECS as of 2019: Michihiro Kamijima^6^ (principal investigator), Shin Yamazaki^7^, Yukihiro Ohya^8^, Reiko Kishi^9^, Nobuo Yaegashi^10^, Koichi Hashimoto^11^, Chisato Mori^12^, Shuichi Ito^13^, Zentaro Yamagata^14^, Takeo Nakayama^15^, Hiroyasu Iso^16^, Masayuki Shima^17^, Youichi Kurozawa^18^, Narufumi Suganuma^19^, Koichi Kusuhara^20^, Takahiko Katoh^21^

^6^ Graduate School of Medical Sciences Department of Occupational and Environmental Health, Nagoya City University, 1 Kawasumi, Mizuho-cho, Mizuho-ku, Nagoya, Aichi 467-8601, Japan. E-mail: [jecscore@nies.go.jp](mailto:jecscore@nies.go.jp)

^7^ National Institute for Environmental Studies, Tsukuba, Japan

^8^ National Center for Child Health and Development, Tokyo, Japan

^9^ Hokkaido University, Sapporo, Japan

^10^ Tohoku University, Sendai, Japan

^11^ Fukushima Medical University, Fukushima, Japan

^12^ Chiba University, Chiba, Japan

^13^ Yokohama City University, Yokohama, Japan

^14^ University of Yamanashi, Chuo, Japan

^15^ Kyoto University, Kyoto, Japan

^16^ Osaka University, Suita, Japan

^17^ Hyogo College of Medicine, Nishinomiya, Japan

^18^ Tottori University, Yonago, Japan

^19^ Kochi University, Nankoku, Japan

^20^ University of Occupational and Environmental Health, Kitakyushu, Japan

^21^ Kumamoto University, Kumamoto, Japan
